# To clip or not to clip the breast tumor bed? A retrospective look at the geographic miss index and normal tissue index of 110 patients with breast cancer

**DOI:** 10.4274/jtgga.2016.0222

**Published:** 2017-06-01

**Authors:** Florian Ebner, Nikolaus de Gregorio, Andreas Rempen, Peter Mohr, Amelie de Gregorio, Achim Wöckel, Wolfgang Janni, Gerlo Witucki

**Affiliations:** 1 Department of Obstetrics and Gynecology, University of Ulm, Ulm, Germany; 2 Department of Obstetrics and Gynecology, Diakonie-Klinikum Schwäbisch Hall, Women’s Clinic with Breast Center and Genital Cancer Center, Schwäbisch Hall, Germany; 3 Department of Radiotherapy, Diakonie-Klinikum Schwäbisch Hall, Schwäbisch Hall, Germany; 4 University of Würzburg Head of Department Prof. A. Wöckel Women’s Clinic and Polyclinic, Würzburg, Germany

**Keywords:** Breast cancer, clips, Radiotherapy, geographic miss index, normal tissue index, boost, reduction

## Abstract

**Objective:**

Planning of breast radiation for patients with breast conserving surgery often relies on clinical markers such as scars. Lately, surgical clips have been used to identify the tumor location. The purpose of this study was to evaluate the geographic miss index (GMI) and the normal tissue index (NTI) for the electron boost in breast cancer treatment plans with and without surgical clips.

**Material and Methods:**

A retrospective descriptive study of 110 consecutive post-surgical patients who underwent breast-conserving treatment in early breast cancer, in which the clinical treatment field with the radiologic (clipped) field were compared and GMI/NTI for the electron boost were calculated respectively.

**Results:**

The average clinical field was 100 mm (range, 100-120 mm) and the clipped field was 90 mm (range, 80-100 mm). The average GMI was 11.3% (range, 0-44%), and the average NTI was 27.5% (range, 0-54%). The GMI and NTI were reduced through the use of intra-surgically placed clips.

**Conclusion:**

The impact of local tumor control on the survival of patients with breast cancer is also influenced by the precision of radiotherapy. Additionally, patients demand an appealing cosmetic result. This makes “clinical” markers such as scars unreliable for radiotherapy planning. A simple way of identifying the tissue at risk is by intra-surgical clipping of the tumor bed. Our results show that the use of surgical clips can reduce the diameter of the radiotherapy field and increase the accuracy of radiotherapy planning. With the placement of surgical clips, more tissue at risk is included in the radiotherapy field. Less normal tissue receives radiotherapy with the use of surgical clips.

## INTRODUCTION

**Figure 1:**
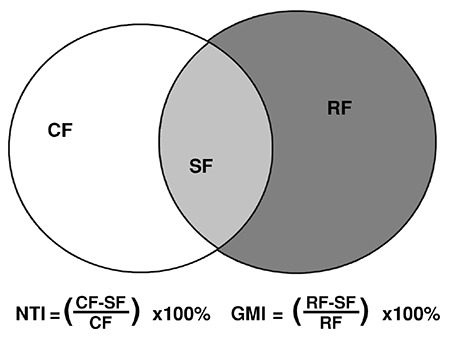
Geographic miss index (GMI) and normal tissue index (NTI): Clinical field (CF); shared field (SF); radiological field (RF)

Wide local excision is the current surgical treatment for most early breast cancers. With the oncologic benefit taken for granted, the cosmetic results are becoming more important (1). In today’s practice, surgeons 'hide' scars around the areola, laterally in the lower axilla or underneath the breast. Guidelines recommend that breast conserving surgery is accompanied by whole breast irradiation. The benefit of guideline-adherent radiotherapy has been clearly demonstrated (2, 3, 4, 5); however, clinical 'landmarks' (i.e. scars) for radiotherapy treatment planning are becoming less reliable. Therefore, the use of surgical clips has been discussed in the last decade (6, 7, 8, 9). Though practical, the use of clips has not been established routinely in some centers, as such proof for the dosimetric advantage is still pending. To estimate the accuracy of radiotherapy treatment, the geographic miss index (GMI) and the normal tissue index (NTI) for the electron boost are used (Figure 1). Ideally, the GMI and the NTI should be as low as possible.

## MATERIAL AND METHODS

Between November 2008 and December 2010, 110 patients with breast cancer who underwent breast conserving surgery with intra-mammary clips and axillary lymph node dissection (ALND) or sentinel node biopsy (SNB) were treated at the Breast Centre Radiotherapy Department with Adjuvant Radiotherapy.

To determine the GMI and NTI in our Breast Cancer Centre, we retrospectively analyzed the radiotherapy treatment plans of 110 patients who underwent breast conserving surgery followed by radiotherapy between 2008 and 2010.

### Statistical analysis

GMI is defined as the percentage of the radiologically-defined field (RF) that is not predicted using clinical landmarks [shared field=SF; GMI=(RF-SF)/RF]. This area represents tissue within the tumor bed, at high risk of local recurrence, which would not have been included in a clinically-marked electron boost field. NTI measures the percentage of the clinically-marked field (CF) that is not part of the RF ('simulation' field'), which receives high-dose treatment [NTI=(CF-SF)/CF].

In our standard surgical protocol, at least three clips are inserted at the margins of the excision cavity and additionally in areas of tumor extension. The volume that the clips cover encloses the former tumor volume. The walls of the excision cavity are approximated at the time of surgery.

Descriptive statistics were calculated with SPSS for Windows (IBM Corp. Released 2010. IBM SPSS Statistics for Windows, Version 19.0. Armonk, NY: IBM Corp) and are given as means, standard deviation (SD), minimum (Min) and maximum (Max).

### Radiation therapy

External beam radiotherapy was planned four to six weeks after completion of chemotherapy or surgery, depending on the clinical situation. Postoperative radiation is given by using a linear accelerator (Elekta Precise) from two (up to four) opposed tangential breast fields, thereby providing a cumulative radiation dose of 50 Gy photons as recommended by the International Commission on Radiation Units&Measurement (10). Mixed energies of 6- and 10-MV photons were used in patients with large breasts. The therapy was administered over a five-week period using 2-Gy daily fractions and a wedge compensator to achieve a uniform dose. The planned target volume encompassed the entire ipsilateral breast. Photon radiation of the entire breast was followed by an electron boost, usually delivering an additional dose of 10 Gy, also in 2-Gy daily fractions.

Clinical markers (scar, memory of patient, hematoma) were used to plan a clinical field area for the electron boost. A 100-mm diameter metal ring was then placed on the breast. X-ray imaging was used to show the clips. On a treatment plan simulation, the clips were outlined and a 30-mm margin was added. The RF ring was then placed around this window. The diameter was taken and the GMI and NTI were calculated.

The study is a descriptive study for standard treatment and did not require ethical approval.

## RESULTS

A total of 110 consecutive patients who underwent breast conserving surgery were included in the study. The average age was 58 years (28-87 years). The average tumor diameter was known in 97.3% of cases. One patient had a complete remission under neoadjuvant treatment and two patients’ final histology data were missing from the database. The diameters ranged from 3 to 52 mm (average 19 mm). After surgery, 75 patients were classified as T1, 31 as T2, two patients had a T4b, and a further two had ductal carcinoma in situ (DCIS). All patients completed the surgical treatment prior to radiotherapy. Ninety-three patients had positive hormone receptors (16 negative) and 19 had herceptin receptor over expression (86 negative, five unknown; further details are provided in Table 1).

The average follow-up was 41 months (30-57 months). One patient had a local recurrence, two had local and distant recurrence, and two had distant recurrences. Of these patients, two died of a tumor-related cause (distant metastasis). One patient died unrelated to the tumor diagnosis (traffic accident). One patient was diagnosed as having contra lateral breast cancer after 47 months. One hundred six patients had hormone therapy +/- chemotherapy. Two patients had a large (>50 mm), high-grade DCIS and therefore hormonal or chemotherapy was not recommended. No further treatment information was available for two cases. After excluding these 4 patients from further analysis, the average clinical field was 100 mm (range, 100-120 mm) and the radiologic field was 90 mm (range, 80-100 mm). The average GMI was 11.3% (range, 0-44%), and the average NTI was 27.5% (range, 0-54%) (see Table 2).

## DISCUSSION

Local disease control is associated with overall survival (11). Efforts have been made to reduce the rate of local recurrence with surgical, systemic therapy, and radiotherapy (12). The influence of systemic therapy on local and distant recurrence has been accepted (13, 14). The surgical resection margin has also been identified as a marker for recurrence rates and the influence of boost radiation (15, 16). The accuracy of the boost can be judged by the GMI and NTI. These indices measure the accuracy of radiotherapy towards the tumor bed. Traditionally, the surgical scar has been used to locate the tumor bed, but breast surgeons and radiation oncologists (17) are becoming more and more concerned about the cosmetic results of their surgery. This results in a scar being a very poor clinical marker for tumor location (6, 18). Patients memory regarding the tumour location is also variable. Fifteen (14%) of our patients had a GMI of 25% or more. This number was lower than the GMI published by Harrington et al. (19). One of the reasons might be the surgical technique of placing the incision immediately over the tumor, which is the common approach of our breast surgeons. Harrington et al. (19) published a GMI depending on the margins between 32.9% (1-cm margin) and 18.6% (3-cm margin) and gave an NTI between 14.6% and 9.7%. Kirby et al. (20) had a GMI of 37% and an NTI of 9%. Twenty-seven cases had a GMI of 0%, meaning that the 'simulation' field was completely covered by the clinical field. With a smaller diameter, the radiologic field resulted in more accurate targeting. In our case series, the NTI was 0% in two patients, with an NTI on average of 27.5%. This shows that even with a good clinical field, one third of high-risk tissue might be missed.

In addition to the above discussion of GMI and NTI, which is based on 2D radiographs, clips offer a further advantage. The dose distribution of the electron boost can be calculated on the basis of computerized tomography (CT) images and 3D planning software. The visibility of the clips allows to select the optimum electron energy that is high enough to cover the clips, but as low as possible to minimize the dose in the lung.

The German Society of Radiooncology practical guidelines for radiotherapy of Breast Cancer I (21) gave the option of placing intra-operative clips, and additionally using presurgical mammography and CT-scans or ultrasound to locate the tumor bed. In the current version, no recommendation is published (22). The S3 guidelines recommend the use of intra surgical clips (13).

One of the limitations of our study is the use of 'classic' external beam radiotherapy. Though it is still commonly in use, the forefront in radiotherapy is shorter treatment protocols, intra-surgical radiation and others (23). Also, it needs to be considered that the intention of the paper was not to provide information on disease-free survival even though the number of patients was fair, but to show the necessity of marking the tumor bed with clips in order to make radiotherapy more precise. The follow-up time was only adequate for early relapses.

Our data clearly demonstrate that with the use of clips in CT radiotherapy planning, the diameter of the field can be reduced by 10 mm on average while increasing the accuracy of the radiotherapy treatment compared with clinical placement with a larger diameter. We think that this can be stated even with such a small case series. Despite this, the common use of intra-surgical clips is not yet established.

## Figures and Tables

**Table 1:**
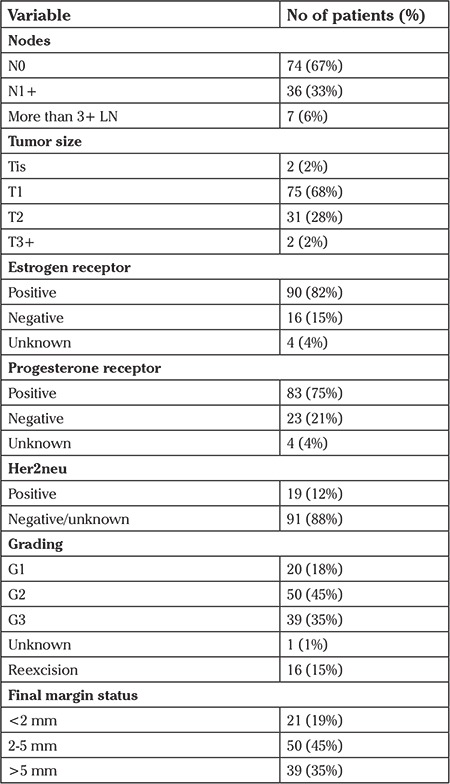
Overview of the tumor data, resection margins and lymph nodes, TNM classification used for tumor size (T), node involvement (N), and grading (G)

**Table 2:**
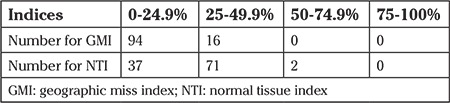
Patients miss indices in quartile distribution
